# Markers for obese and non-obese Type 2 diabetes identified using whole blood metabolomics

**DOI:** 10.1038/s41598-023-29619-4

**Published:** 2023-02-11

**Authors:** Takayuki Teruya, Sumito Sunagawa, Ayaka Mori, Hiroaki Masuzaki, Mitsuhiro Yanagida

**Affiliations:** 1 G0 Cell Unit, Okinawa Institute of Science and Technology Graduate University (OIST), Okinawa, Japan; 2 Division of Endocrinology, Diabetes and Metabolism, Hematology, Rheumatology (Second Department of Internal Medicine), Graduate School of Medicine, University of the Ryukyus, Okinawa, Japan; 3 Present Address: R&D Cluster Programs Section, Technology Development and Innovation Center, Okinawa Institute of Science and Technology Graduate University (OIST), Okinawa, Japan; 4 Present Address: Cell Division Dynamics Unit, Okinawa Institute of Science and Technology Graduate University (OIST), Okinawa, Japan

**Keywords:** Metabolomics, Biochemistry, Biomarkers

## Abstract

Definitive differences in blood metabolite profiles between obese and non-obese Type 2 diabetes (T2D) have not been established. We performed an LC–MS-based non-targeted metabolomic analysis of whole blood samples collected from subjects classified into 4 types, based on the presence or absence of obesity and T2D. Of the 125 compounds identified, 20, comprising mainly nucleobases and glucose metabolites, showed significant increases or decreases in the T2D group. These included cytidine, UDP-glucuronate, UMP, 6-phosphogluconate, and pentose-phosphate. Among those 20 compounds, 11 enriched in red blood cells (RBCs) have rarely been studied in the context of diabetes, indicating that RBC metabolism is more extensively disrupted than previously known. Correlation analysis revealed that these T2D markers include 15 HbA1c-associated and 5 irrelevant compounds that may reflect diabetic conditions by a different mechanism than that of HbA1c. In the obese group, enhanced protein and fatty acid catabolism causes increases in 13 compounds, including methylated or acetylated amino acids and short-chain carnitines. Our study, which may be considered a pilot investigation, suggests that changes in blood metabolism due to obesity and diabetes are large, but essentially independent.

## Introduction

Human activities are enabled by blood. Blood carries oxygen and numerous nutrients and eliminates wastes. Hundreds of blood metabolites are documented and many are markers for health and disease. Critical health-related information about physiological processes is encoded in titers of individual blood metabolites^1,2^. Because blood samples are easily collected, blood metabolites can be exploited for detailed and thorough studies of various diseases, including diabetes. Type 2 diabetes (T2D) occurs when the body does not produce enough insulin, or when the cells cannot use insulin properly, a state known as insulin resistance. If insulin production is insufficient, excess glucose remains in the blood, resulting in hyperglycemia. In Type 1 diabetes, insulin is not produced, a condition known as juvenile diabetes. In contrast, T2D is a lifestyle disease, appearing later in life. T2D accounts for 90–95% of all cases of diabetes and its development is affected by genetic and environmental factors. If hyperglycemia continues over a long period, micro-capillaries are damaged, causing complications such as heart disease, retinopathy, nephropathy, neurological disorders, etc. Therefore, treating and preventing T2D are global priorities and metabolic markers that indicate changes in metabolism portending the onset of T2D are needed. Higher blood sugar results in more glycosylated hemoglobin. The blood concentration of HbA1c is an index that reflects the average blood sugar level over past several months^3^. Furthermore, diabetes-induced chronic hyperglycemia results in formation of advanced glycation end products (AGEs), a heterogeneous group of non-enzymatically derived glycosylation products of proteins and lipids^4^. Recent studies have shown an association between cutaneous accumulation of advanced glycation end products and cardiovascular risk in patients with T2D^5^.The worldwide explosion of T2D has been attributed primarily to the increased incidence of obesity. On the other hand, T2D with lean/normal body mass index (abbreviated BMI), which once comprised a minority of diabetic cases, has recently increased explosively in Asia and other countries^6–9^. However, causes and mechanisms of T2D without obesity are much less well understood than those of T2D with it^2^. It is believed that genetic, acquired, and behavioral factors underpin development of diabetes, but due to its complexity, the principal metabolic processes resulting in diabetes remain enigmatic.

In this study we conducted non-targeted, accurate semi-quantitative metabolomic analysis that provides complete information about metabolite species and their abundances in each subject. In contrast, metabolites analyzed in targeted analysis, are pre-determined so that most changes in abundance may be overlooked. While non-targeted analysis is far more time-consuming, the effort expended in this “no assumptions” approach is very often rewarded with new discoveries of changes in abundance of crucial compounds. Accordingly, we discovered a number of blood compounds that alter their abundance in obesity and diabetes.

Previous T2D metabolomic studies have focused mainly on obese subjects, and few studies have examined lean or normal-weight T2D subjects. In those studies, metabolite analysis was conducted mainly on plasma, so that RBC metabolites were generally not quantified. The present study emphasizes whole blood metabolomics of T2D subjects with and without obesity, with special attention to metabolite titers in red blood cells (RBCs). This novel approach revealed unexpected responses of metabolites enriched in RBCs, including those of oxido-reductants, anti-oxidants, sugar phosphates, and nucleotides^10^. We measured abundances of blood metabolites in four groups of subjects, obese or non-obese and T2D or non-T2D, and identified metabolites related to diabetes and/or obesity. Although our study is highly detailed regarding metabolite species and abundance, this is an early, pilot study, and statistically robust conclusions will require investigation of far larger numbers of subjects (hundreds or thousands). However, some of these results corroborate those of previous studies.

## Results

### Sample collection and characteristics of subjects

**Figure 1:**
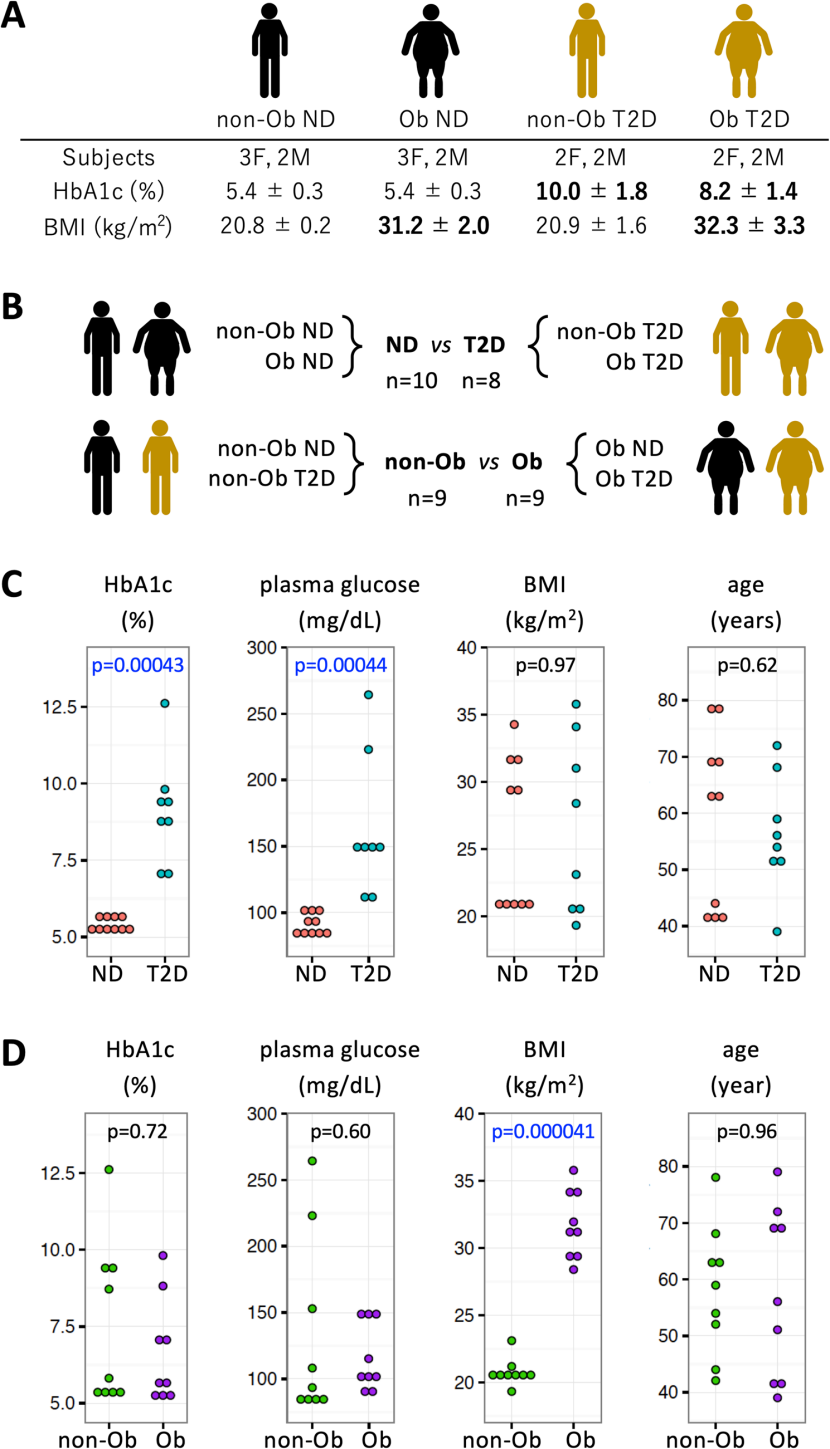
Characteristics and classification of 18 subjects. (**A**) Subjects were divided into four groups based on their HbA1c levels and BMI. Gender composition, mean ± SD of HbA1c, and BMI for each group are shown. (**B**) Group composition of non-diabetic (ND), T2D, non-obese (non-Ob), and obese (Ob) groups. (**C,D**) Graphic presentations of HbA1c, plasma glucose, BMI, and age in ND vs T2D (**C**) and non-Ob vs Ob (**D**). In each panel, p-values are presented to show the significance of differences. P-values less than 0.05 are highlighted in blue.

Whole blood samples were obtained from four groups of subjects: 1. Non-obese, non-diabetic (*non-Ob ND*)*,* 2. Obese, non-diabetic (*Ob ND*)*,* 3. Obese, diabetic (*Ob T2D*)*,* 4. Non-obese, diabetic (*Non-Ob T2D*). Eighteen subjects comprising 5 non-Ob ND subjects, 5 Ob ND subjects, 4 Ob T2D subjects, and 4 non-Ob T2D subjects participated in this study (Fig. 1A). Blood samples were obtained from each T2D patients (HbA1c 6.9 to 12.6%), diagnosed and hospitalized at the University of the Ryukyus Hospital, in Nishihara, Okinawa. Non-diabetic healthy volunteers (HbA1c 5.1 to 5.8%) from Onna Clinic, in Onna, Okinawa, were also recruited. Characteristics of each subject (age, gender, BMI, HbA1c, plasma glucose level) are shown in Supplementary Table S1. When subjects were divided into ND and T2D, there was a significant difference in HbA1c and plasma glucose between them, but no significant difference in BMI or age (Fig. 1B,C). In contrast, when subjects were divided into non-Ob and Ob, there was a significant difference in BMI, but no significant difference in HbA1c, plasma glucose, or age (Fig. 1B,D). These data show that each combination is appropriate for unbiased identification of diabetes and obesity-related metabolites.

### Quantification of blood metabolites

To identify metabolites affected by T2D or obesity, semi-quantitative metabolomic analysis was conducted using non-targeted LC–MS. To ensure reproducible results, all blood samples for metabolomic analysis were quenched at – 40 °C immediately after blood collection. Procedures of LC–MS analysis, compound identification, and data processing with MZmine 2 were done as previously reported^10,11^. We identified and quantified 125 whole blood metabolites for all subjects (Supplementary Table S2), and these were classified into 14 subgroups (Supplementary Table S3). Metabolites included 9 nucleotides, 14 nucleosides, nucleobases, 5 vitamins and coenzymes, 4 nucleotide-sugar derivatives, 11 sugar phosphates, 4 sugar derivatives, 6 lipid metabolites, 10 carnitines, 9 organic acids, 2 antioxidants, 17 standard amino acids, 13 methylated amino acids, 4 acetylated amino acids, and 17 other amino acids. Fifty-three metabolites were enriched in RBCs, constituting ~ 42% (52/125) of whole blood metabolites^10,12^.

### 20 metabolites are T2D-linked

**Figure 2:**
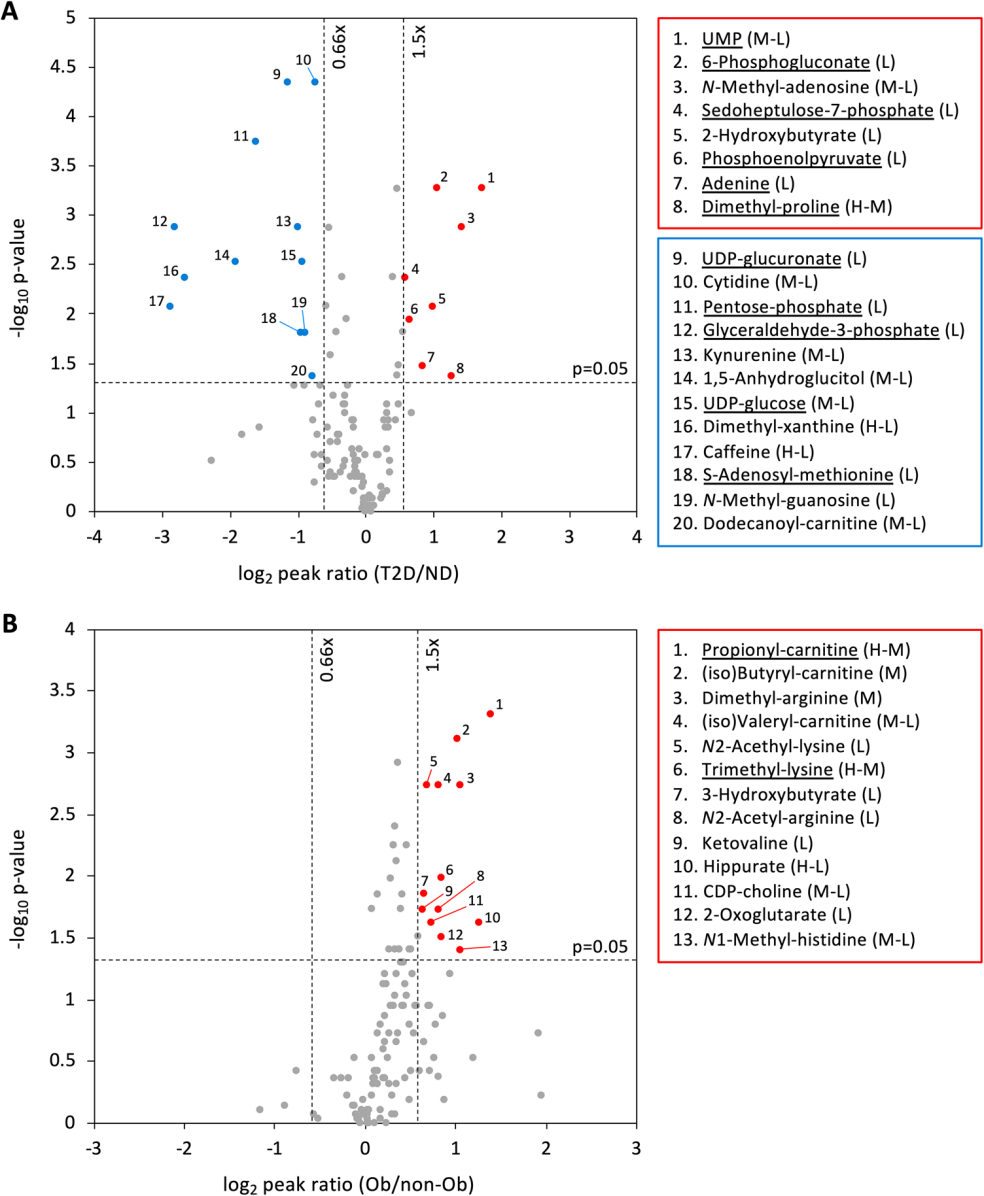
Volcano plot analysis of blood metabolites. The identified 125 compounds are plotted. The x-axis is the log2-fold change, whereas the y-axis is the -log10 p value of the Mann–Whitney U-test. Two vertical dashed lines indicate 0.66 and 1.5-fold changes. The horizontal dashed line shows a p value = 0.05. (**A**) Metabolites that were more than 1.5 times higher in T2D than ND are shown in red, and metabolites that were less than two-thirds (0.66 times) lower are shown in blue. (**B**) Similar to (**A**), metabolites that were more than 1.5 times higher in Ob than non-Ob are shown in red, and metabolites that were less than two-thirds (0.66 times) lower are shown in blue. The highlighted 20 compounds in (**A**) and 13 in (**B**) were identified as T2D markers and obesity markers, respectively. RBC-enriched compounds are underlined.

T2D-related metabolites were compared between ND and T2D. Statistical analysis was performed using the non-parametric Mann–Whitney *U* test. A peak ratio was calculated using the median of peak abundance in the ND and T2D groups. Of the 125 compounds, 20 metabolites differed significantly between ND and T2D subjects (p < 0.05, peak ratio (T2D/ND) < 0.66 or > 1.5) (Fig. 2A and Supplementary Table S3). These 20 metabolites comprise 1 nucleotide (UMP), 6 nucleosides and nucleobases (cytidine, *N*-methyl-adenosine, *N*-methyl-guanosine, adenine, caffeine, dimethyl-xanthine), 2 nucleotide-sugars (UDP-glucose, UDP-glucuronate), 5 sugar phosphates (6-phosphogluconate^13^, glyceraldehyde-3-phosphate, pentose-phosphate, phosphoenolpyruvate^14^, sedoheptulose-7-phosphate), 1 sugar derivative (1,5-anhydroglucitol^15,16^), 1 carnitine (dodecanoyl-carnitine^17^), 1 organic acid (2-hydroxybutyrate^18,19^), 1 methylated amino acid (dimethyl-proline), and 2 other amino acids (*S*-adenosyl-methionine, kynurenine). Eight of the 20 compounds (Fig. 2A) increased in T2D, whereas 12 compounds decreased. 1,5-Anhydroglucitol, known as a glycemic marker^20^, showed strikingly lower levels in T2D patients than in ND subjects (p = 0.0031, Supplementary Fig. S1). The distribution of UDP-glucuronate and cytidine most clearly separated ND and T2D (p = 0.000046). The median value of caffeine in both groups showed the largest difference (peak ratio, T2D/ND = 0.14) among the 20 compounds, but the p-value was not so low due to large individual differences. Statistical power is shown in Supplementary Table S3. Thirteen of 20 T2D markers exceeded the power of 0.8 for a statistical test to be valid according to Cohen’s logic^21^. Levels of blood compounds differed greatly. The peak area value difference was nearly 1,000-fold, for example, between caffeine or dimethyl-proline (10^8^ AU) and *N*-methyl-guanosine (10^5^ AU) (Supplementary Fig. S1). However, peak area values (10^5^ ~ 10^9^ AU) require calibration to convert them to molar concentrations^22^. Eleven of the 20 compounds are enriched in RBCs. To our knowledge, 9 compounds abundant in RBCs (UMP, adenine, UDP-glucose, UDP-glucuronate, glyceraldehyde-3-phosphate, pentose-phosphate, sedoheptulose-7-phosphate, dimethyl-proline, *S*-adenosyl-methionine) and 4 compounds present in both plasma and RBC (cytidine, caffeine, dimethyl-xanthine, kynurenine), have not previously been reported as diabetes markers. These results show that glucose metabolites enriched in RBCs, which are of relatively low abundance in whole blood, and nucleobase derivatives present in both plasma and RBCs are the metabolites most affected by diabetes.

### 13 metabolites increased in obese subjects

When non-Ob and Ob subjects were compared, thirteen metabolites, comprising 3 carnitines (propionyl-carnitine, butyryl-carnitine, valeryl-carnitine)^23–25^, 3 methylated amino acids (*N*1-methyl-histidine, dimethyl-arginine^26,27^, trimethyl-lysine), 2 acetylated amino acids (*N*2-acetyl-arginine, *N*2-acetyl-lysine), 2 organic acids (2-oxoglutarate^23^, 3-hydroxybutyrate^28^), 1 lipid metabolite (CDP-choline), and 2 other amino acids (ketovaline^25^, hippurate), significantly increased in obese subjects (Fig. 2B and Supplementary Table S3). Among them, 3 carnitines (dots no. 1, 2, 4 in Fig. 2B) and dimethyl-arginine and *N*2-acetyl-lysine showed a relatively clear difference between the two groups (p = 0.00049 to 0.0019). All other compounds had p-values from 0.011 to 0.040 and peak ratios (Ob/non-Ob) less than 1.5. Seven of the 13 obesity markers had a statistical power > 0.8 (Supplementary Table S3). No metabolites significantly decreased in obese people. Only 2 of the 13 compounds were abundant in red blood cells. There was no overlap with compounds that showed a significant difference in diabetes. Thus, in contrast to T2D-related metabolites, obesity-related metabolites are primarily characterized by an increase in metabolites that occur during fatty acid degradation and proteolysis.

### Connections of metabolites revealed by Pearson’s correlation coefficients

**Figure 3:**
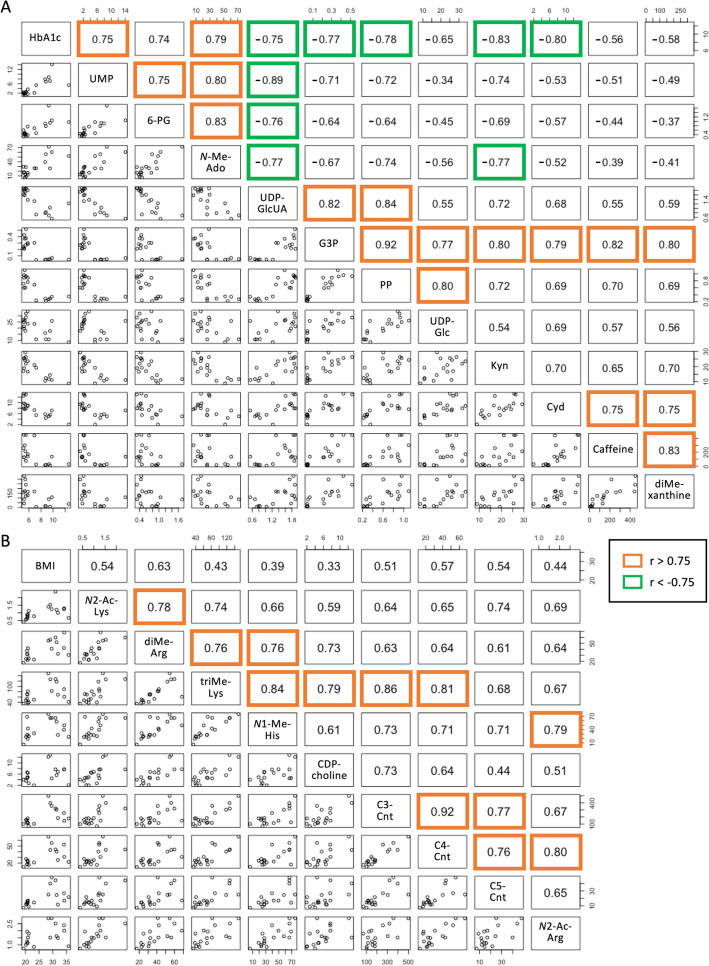
Pearson's correlation analysis identified strong metabolic relationships. (**A**) Correlation coefficients of T2D markers were calculated. Orange and green boxes indicate positive (r > 0.75) and negative (r < − 0.75) correlations, respectively. *6-PG* 6-phosphogluconate, *N-Me-Ado* *N*-methyl-adenosine, *UDP-GlcUA* UDP-glucuronate, *G3P* glyceraldehyde-3-phosphate, *PP* pentose-phosphate, *UDP-Glc* UDP-glucose, *Kyn* kynurenine, *Cyd* cytidine, *diMe-xanthine* dimethyl-xanthine. (**B**) Obesity markers with a correlation of 0.75 or higher are shown. *N2-Ac-Lys* *N*2-acetyl-lysine, *diMe-Arg* dimethyl-arginine, *triMe-Lys* trimethyl-lysine, *N1-Me-His* N1-methyl-histidine, *C3-Cnt* propionyl-carnitine, *C4-Cnt* butyryl-carnitine, *C5-Cnt* valeryl-carnitine, *N2-Ac-Arg* N2-acetyl-arginine.

In order to better understand relationships among metabolites, we extended our quantitative analysis using Pearson’s correlation formula for 20 diabetes-linked and 13 obesity-linked metabolites (Supplementary Fig. S3). Of 149 significant correlations, 118 were positive and 31 were negative. Positive correlations were frequently and broadly found among T2D or obesity-linked compound groups, probably because those metabolites belonged to the same or related metabolic pathways and have similar functions. For example, we confirmed correlations between caffeine metabolites, caffeine and dimethyl-xanthine (r = 0.83, p = 0.00002), and between methylated amino acids, dimethyl-arginine and *N*1-methyl-histidine (r = 0.76, p = 0.00025), as previously reported^10,29,30^. Of the 20 T2D-linked compounds, all except dimethyl-xanthine and *S*-adenosyl-methionine had negative correlations. Some nucleotide and sugar metabolites showed strong negative correlation coefficients: UDP-glucuronate and UMP (r = − 0.89, p < 0.00001), UDP-glucuronate and 6-phosphogluconate (r = − 0.76, p = 0.00025), UDP-glucuronate and *N*-methyl-adenosine (r = − 0.77, p = 0.00019). These compounds with negative correlations participate in competing or inhibiting metabolic reactions. Eleven compounds include 5 sugar metabolites (6-phosphogluconate, UDP-glucuronate, glyceraldehyde-3-phosphate, pentose-phosphate, UDP-glucose), 5 nucleobases (*N*-methyl-adenosine, UMP, caffeine, dimethyl-xanthine, cytidine), and kynurenine had a particularly strong correlation of r > 0.75 or r < − 0.75 (p < 0.00034) (Fig. 3A). Interestingly, these 11 metabolites showed significant correlations with HbA1c, but did not correlate with plasma glucose. This suggests that changes in these metabolites are not short-term and are probably associated with relatively long-term hyperglycemic conditions like HbA1c. It is impressive that kynurenine and cytidine showed high correlations with HbA1c at − 0.83 and − 0.80, respectively, but the reasons are not clear. The extensive, strong correlation network appears to reflect characteristics of diabetes caused by cross-sectional impairment in the glycolysis/gluconeogenesis pathway, pentose phosphate pathway, glucuronic acid pathway, and purine/pyrimidine pathway, due to chronic hyperglycemia.

All 13 obesity-related compounds showed positive correlations, with 9 compounds (excluding 2-oxoglutarate, ketovaline, hippurate, and 3-hydroxybutyrate) having r > 0.75 (Supplementary Fig. S3). The correlation between these metabolites and BMI was moderate (r = 0.33 ~ 0.63). No metabolites had correlation coefficients > 0.75 with BMI (Fig. 3B). Some acetylated or methylated-amino acids (*N*2-acetyl-lysine, dimethyl-arginine, trimethyl-lysine, *N*1-methyl-histidine, *N*2-acetyl-arginine) were significantly increased in the obese group and displayed strong positive correlation coefficients within them (r = 0.64 ~ 0.84) and with short-chain acylcarnitines (propionyl-carnitine, butyryl-carnitine, valeryl-carnitine) (r = 0.61 ~ 0.86). Trimethyl-lysine, which is involved in carnitine synthesis, showed a strong correlation (0.79) with CDP-choline, a precursor of glycerophosphocholine. Thus, these results represent the coordinated activation of protein and lipid turnover in obesity.

T2D-linked metabolites and obesity-linked metabolites formed correlated clusters within their respective groups, but no correlation of 0.75 or greater was observed between the two groups. In addition, of the 20 T2D markers, no metabolites showed a correlation of 0.5 or more with BMI, and none of the 13 obesity markers showed correlation coefficients with HbA1c ≥ 0.5. This indicates that changes in blood metabolism due to T2D and obesity are fundamentally different. Whether these metabolites change as a consequence of disease or as a cause of it, remains to be determined.

### Metabolic profile of subjects visualized by heatmap data

A heat map was created to visualize relative levels of T2D and obesity markers in each subject (Fig. 5). Abundances of 20 T2D markers and 13 obesity markers reflected the degree of deviation from average. Metabolites were sorted by cluster analysis based on correlations between metabolites. In diabetic subjects, with or without obesity, 8 metabolites higher in T2D patients mostly indicated over 50 (red) of T-scores due to being above average (50), and 12 compounds lower in diabetes patients mostly showed less than 50 (blue) due to being below average. The non-diabetic group (non-Ob ND and Ob ND) showed the opposite trend to the diabetic group (non-Ob T2D and Ob T2D). Levels of all obesity markers mostly showed blue due to being below average in non-obese subjects, regardless of diabetes. Some obese subjects (no.7, 9, 16) show lower abundances, as in non-obese people. All were females. This suggests that there may be gender differences in metabolic responses to obesity^31^. Further verification is needed. In addition, subject No. 18 had weaker changes in metabolites compared to other diabetic patients. The HbA1c value of this patient was the lowest in the diabetic group, so this patient’s diabetic tendency may be relatively mild. Thus, this heatmap reveals metabolite profiles by subject group, as well as individual differences within the group.

### PCA resolves subjects into four distinct groups

**Figure 4:**
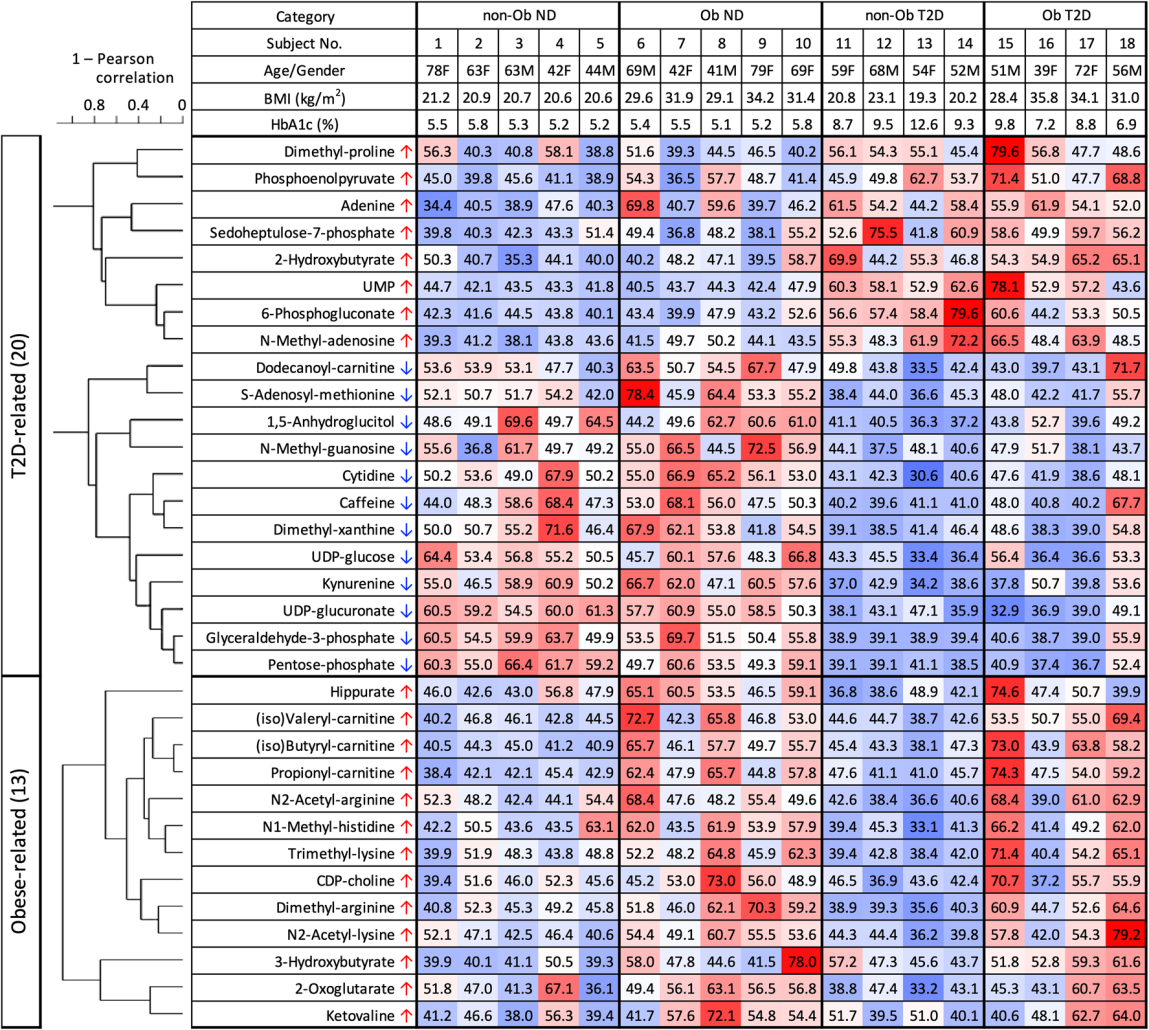
Hierarchical clustering heatmap of 20 T2D and 13 obesity-related compounds. A heat map was constructed for 18 subjects using a color matrix representing relative abundance data of 33 metabolites implicated in diabetes or obesity. Numerical values indicate the T score, a kind of standardized score. The average and standard deviation are 50 and 10, respectively. Blue cells indicate metabolite titers that are below average (50), whereas red cells indicate levels that are higher than average. Color intensity of cells reflects the T score.

**Figure 5:**
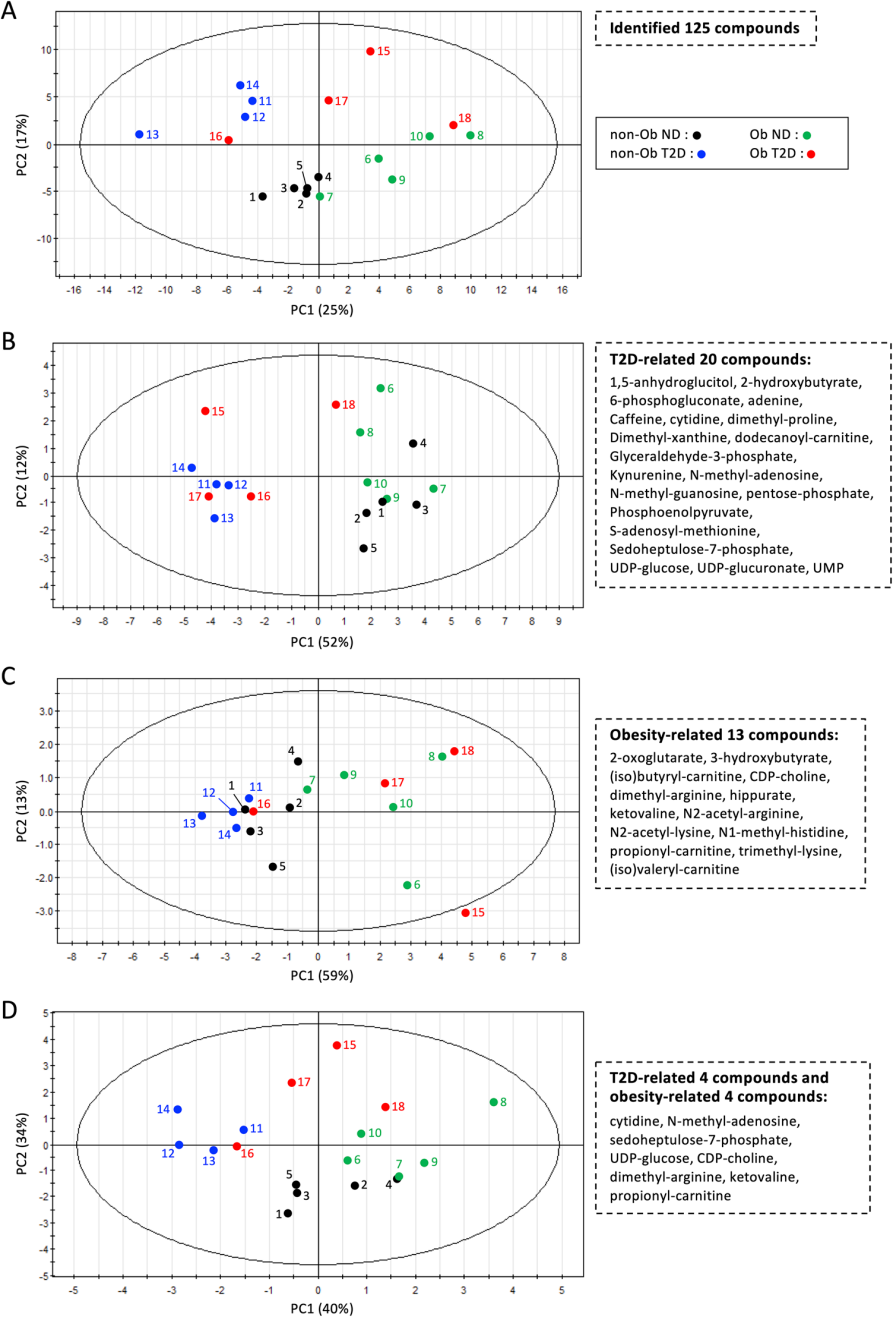
PCA plots of T2D and obesity-related compounds. (**A**) All 125 compounds identified in the present study roughly divided the T2D and ND groups. (**B**) Twenty diabetic markers separated ND and T2D subjects, except one subject (no. 18). (**C**) Thirteen obesity markers largely separated non-Ob and Ob subjects. (**D**) Even eight metabolites separated four subject groups, Ob T2D, non-Ob ND, non-Ob T2D, and Ob ND, to up, down, left, and right sides, respectively.

Quantitative metabolite data were subjected to principal component analysis (PCA) so as to reduce the dimensionality of abundance data for T2D and obesity-linked metabolites. A PCA plot using all 125 compounds roughly divided the T2D and ND groups into the upper left and lower right quadrants, respectively (Fig. 5A). In each cluster, obese subjects are located to the right of non-obese subjects, suggesting that diabetes and obesity are the largest factors (PC1 25% + PC2 17% = 42%) in the variability of metabolite abundances in this dataset. When all 20 diabetic marker metabolites were used for PCA, the distribution of subjects was clearly divided into diabetic and non-diabetic groups, except for subject no.18 (Fig. 5B). No.18 had mild diabetes (Fig. 4), so his marker distribution was closer to those of non-diabetic patients. We then employed 13 obesity markers (Fig. 5C). In that plot, non-obese and obese people were generally located on opposite sides, with some exceptions. All subjects on or near the non-obese side were females. Among obese people, males were to the obese side of females. Given the heatmap patterns (Fig. 4), this distribution is reasonable. Diabetes is more clearly divided than obesity, probably because there are more compounds with significant p-values among diabetes markers (Fig. 2).

We then attempted to reduce the number of metabolites needed to separate the 4 groups of subjects. After many attempts, we found that combinations of as few as 8 metabolites, comprising 4 T2D markers and 4 obesity markers were able to separate the subjects into the 4 rough groups (Fig. 5D). The four T2D markers included cytidine, *N*-methyl-adenosine, sedoheptulose-7-phosphate, and UDP-glucose, while the four obesity markers were CDP-choline, dimethyl-arginine, ketovaline, and propionyl-carnitine.

## Discussion

Although long-term hyperglycemia affects the structure and function of RBCs^32–34^, RBCs are generally not included in metabolomic analysis because they are labile^35,36^. We stabilized these metabolites using a previously reported rapid quenching method, and then analyzed the whole blood extract after removing large molecules^10^. Using whole blood, rather than just blood plasma, a number of novel markers including metabolites specifically enriched in RBCs were discovered^10,11,29,30,37^. Since about 40% of whole blood consists of blood cells, the chances of finding new metabolites were high. Indeed, among 7 previously reported diabetes markers, only 2 (6-phosphogluconate and phosphoenolpyruvate) were enriched in RBCs. We were able to identify 20 diabetic markers, and 11 of them are RBC-enriched (Fig. 2). In contrast, 13 obesity markers included only 2 RBC-enriched metabolites (propionyl-carnitine and trimethyl-lysine). These results suggest that while metabolism in red blood cells is vulnerable to hyperglycemic conditions, it is less susceptible to obesity. RBCs, which do not possess mitochondria, are thought to rely solely on glucose for energy. Glucose transporter 1 (GLUT1) mediates the transmembrane transport of glucose in an insulin-independent manner^38^. When blood sugar levels rise, more glucose enters red blood cells and the glucose metabolic pathway is accelerated accordingly. Therefore, RBCs may be highly susceptible to hyperglycemia.

Eight compounds that increased and 12 compounds that decreased in T2D were significantly inversely correlated (Supplementary Fig. S3). Interestingly, 11 of the 12 compounds that were reduced in T2D, including 1,5-AG, a known T2D marker, were inversely correlated with HbA1c, but not with plasma glucose levels^38,39^. The 4 increased compounds (phosphoenolpyruvate, UMP, 6-phosphogluconate, and *N*-methyl-adenosine) were also correlated with HbA1c. This suggests that these 15 compounds change depending on the glucose concentration in the blood over a period of time, like HbA1c. Pentose-phosphate, 6-phosphogluconate, and glyceraldehyde-3-phosphate are metabolites of the glycolysis/pentose phosphate pathway, and UDP-glucose, UDP-glucuronate, and UMP are metabolites of the uronic acid pathway. These metabolites showed strong positive and negative correlations (Fig. 3). Since both pathways originate from a glycolytic intermediate glucose-6-phosphate, these metabolic pathways may compete for glucose utilization in diabetics. However, further research is needed to understand why compounds such as *N*-methyl-adenosine and kynurenine, which are structurally and functionally different, correlate with their glucose metabolites. On the other hand, the remaining 5 compounds (dimethyl-proline, adenine, sedoheptulose-7-phosphate, 2-hydroxybutyrate, *N*-methyl-guanosine), which did not show a significant correlation with HbA1c, are probably changed by mechanisms different from that which affects HbA1c, but this mechanism is not easy to infer. It will be worth investigating how these metabolic changes in RBCs affect diabetes and its complications.

The 13 compounds that increased in the obesity group were positively correlated with each other. *N*2-acetyl-lysine and *N*2-acetyl-arginine are produced by degradation of N-terminally acetylated proteins^40^. No N-terminal deacetylase has been discovered, so N-terminal acetylation is thought to be irreversible. Dimethyl-arginine and trimethyl-lysine are degradation products of histone and non-histone proteins^41,42^. Little is known about the human protein from which *N*1-methyl-histidine is derived^43^. An increase in these modified amino acids may be due to activation of protein metabolism and/or decreased urinary excretion in obese individuals. Three acylcarnitines (propionyl-carnitine, butyryl-carnitine, and valeryl-carnitine) are involved in beta-oxidation of fatty acids in mitochondria. These are called short-chain acylcarnitines and are synthesized not only from fatty acids, but also from the branched-chain amino acids, leucine, isoleucine, and valine. Ketovaline and 3-hydroxybutyrate are metabolites belonging to the ketone body synthesis pathway, which is an alternative energy source for glucose. CDP-choline is a precursor of phosphatidylcholine. 2-Oxoglutarate is a metabolite of the citric acid cycle. Hippurate is a food-derived benzoic acid metabolite. These observations are consistent with previous reports^23,24,44,45^. Taken together, the increase in these compounds suggests that in obese people, turnover of proteins, lipids, and other high-energy compounds is activated^45–47^.

The degree of metabolic changes due to T2D and obesity are visually represented with a heatmap and PCA. The heatmap shows not only the metabolic pattern of each subject group, but also the degree of change in each metabolite in each individual (Fig. 4). In contrast, PCA reduces the dimensionality of metabolite data and simply represents similarities of marker profiles between individuals by distance between dots (Fig. 5). Blood levels of 12 compounds decreased in T2D in subject no.18 were similar to those of NDs (Fig. 4). For that, this individual appeared on the ND side in PCA (Fig. 5B). Note that these aforementioned 12 compounds have a strong correlation with HbA1c (Fig. 3A) and that this subject's HbA1c was the lowest in the T2D group (Fig. 4). On the other hand, subject no.13, who had the lowest BMI among all subjects, showed an overall low level of obesity-related metabolites (Fig. 4). PCA using obesity marker data gave subject 13 the lowest PC1 score, indicating the lowest propensity for obesity (Fig. 5C). Thus, a broad overview and detailed quantification of metabolites are useful to grasp the effects of diabetes and obesity on blood metabolism. Our results suggest that changes in blood metabolites due to obesity and diabetes are independent. Hitherto, this has not been emphasized in diabetes studies. These findings not only illuminate diabetes pathology, but also serve to guide development of future prevention and treatment methods.

## Methods

### Blood donors

Eight T2D patients, hospitalized at Ryukyu University Hospital, in Nishihara, Okinawa, participated as subjects. In addition, ten healthy volunteers who live in Onna Village, Okinawa, participated as volunteers. Subjects were evaluated for group membership: non-obese, non-T2D (non-Ob ND); obese, non-T2D (Ob T2D); non-obese with T2D (non-Ob T2D), and obese with T2D (Ob T2D) by measuring diabetes metabolic markers such as HbA1c, BMI, etc. The Non-Ob ND group fell within the normal weight range (BMI = 20.6 ~ 21.1), while the Ob ND group had BMI = 29.1 ~ 34.2. Age (39 ~ 79 yr), gender (10 females and 8 males), HbA1c values (ND 5.1 ~ 5.8; T2D 6.9 ~ 12.6%), and fasting plasma glucose levels (ND 81 ~ 102; T2D 108 ~ 264 mg/dL) are provided in Supplementary Fig. S1. The two diabetic groups (non-Ob T2D and Ob T2D) differed significantly in regard to BMI (non-Ob T2D, 19.3 ~ 23.1 vs Ob T2D 28.4 ~ 35.8).

### Clinical assessment

In this study, a BMI of 25 kg/m^2^ or more was defined as obesity, according to criteria set by the Japan Obesity Society. In Japan, the proportion of people with advanced obesity is lower than in other countries, and a cohort study in Japan reported that mild obesity can easily lead to health problems^48^. Diabetes was diagnosed based on guidelines published by the Japanese Diabetes Society. These guidelines use the National Glycohemoglobin Standardization Program (NGSP) values of HbA1c^49^.

### Ethics statement

Written, informed consent was obtained from all donors in accordance with the Declaration of Helsinki. All experiments were performed in compliance with relevant Japanese laws and institutional guidelines. All protocols were approved by the institutional ethics committee of the University of the Ryukyus (approval number: 382) and the Human Subjects Research Review Committee of the Okinawa Institute of Science and Technology Graduate University (OIST) (approval number: HSR-2011-003).

### Sample preparation

Metabolomic samples were prepared as reported previously^10,12^. Subjects fasted overnight and blood samples were taken before breakfast. During that time, subjects were allowed to drink water freely. All blood samples were drawn in a hospital laboratory to ensure rapid sample preparation. Blood samples from diabetic subjects were collected at Ryukyu University Hospital, Nishihara, Okinawa, while non-diabetic blood samples were obtained from healthy volunteers at the Onna Clinic, Onna Village, Okinawa. Briefly, venous blood samples for metabolomic analysis were collected in 5-mL heparinized tubes (Terumo, Tokyo, Japan). Immediately, 0.2 mL blood were quenched in 1.8 mL 50% methanol at − 40 °C. This quenching immediately after blood sampling ensured accurate measurement of many labile metabolites. Use of whole blood samples also allowed us to examine blood cellular metabolite levels that might otherwise have been affected by lengthy cell separation procedures. Two internal standards, 10 nmol each of 4-(2-hydroxyethyl)-1-piperazineethanesulfonic acid (HEPES) and piperazine-*N*, *N*’-bis (2-ethanesulfonic acid) (PIPES) were added to each sample as a reference. After brief vortexing, samples were transferred to Amicon Ultra 10-kDa cut-off filters (Merck Millipore, Billerica, MA, USA) to remove proteins and cellular debris. After sample concentration by vacuum evaporation, each sample was re-suspended in 40 µL of 50% acetonitrile, and 1 µL was used for each injection into the LC–MS system.

### Chemicals and reagents

Standards for metabolite identification were purchased from commercial sources, as described previously^11,12^. All solvents and ultrapure water were of LC–MS grade (FUJIFILM Wako Pure Chemical Corporation, Osaka, Japan).

### Non-targeted LC–MS analysis

Non-targeted LC–MS data were obtained using an Ultimate 3000 DGP-3600RS (Thermo Fisher Scientific, Waltham, MA, USA) coupled to an LTQ Orbitrap mass spectrometer (Thermo Fisher Scientific, Waltham, MA, USA), as previously described^10–12^. Briefly, LC separation was performed on a ZIC-pHILIC column (150 mm × 2.1 mm, 5 µm particle size; Merck Millipore, Burlington, MA, USA). The HILIC column is quite useful for separating many hydrophilic blood metabolites that are generally not assayed^50^. Acetonitrile (A) and 10 mM ammonium carbonate buffer, pH 9.3 (B) were used as the mobile phase, with gradient elution from 80 to 20% A in 30 min, at a flow rate of 100 µL/mL. An electrospray ionization (ESI) source was used for MS detection. Each sample was injected twice (1 µL volume/injection), one with the ESI operated in negative ionization mode and the other in positive ionization mode. Spray voltage and capillary temperature were set to 2.8 kV (negative ESI) or 4.0 kV (positive ESI) and to 350 °C or 300 °C, respectively. Nitrogen was used as the carrier gas. The mass spectrometer was operated in a full scan mode with a 100–1000 m/z scan rage and automatic data-dependent MS/MS fragmentation scans. Peak areas of metabolites of interest were measured using MZmine 2 software^51,52^. Peak identification was based on retention time and parent and fragment masses (m/z) followed by quantification with relative peak intensity.

### Peak identification and characteristics

We analyzed 125 blood compounds that were confirmed using standards or MS/MS analysis^10–12^. For each metabolite we chose a singly charged, [M+H]^+^ or [M-H]^-^, peak. Metabolites were classified into 3 groups (H, M, and L), according to their peak areas. H denotes compounds with high peak areas (> 10^8^ AU), M with medium peak areas (10^8^ ~ 10^7^ AU) and L with low peak areas (< 10^7^ AU) (Supplemental Table S2).

### Statistical analysis

Data processed using MZmine 2 were exported to a spreadsheet and dot plots were drawn with R statistical software (http://www.r-project.org). Compounds related to diabetes or obesity were identified with the non-parametric Mann–Whitney U-test. Statistical significance was set at p < 0.05. A post-hoc power analysis was conducted with G*Power 3.1.9.6 (https://www.psychologie.hhu.de/arbeitsgruppen/allgemeine-psychologie-und-arbeitspsychologie/gpower). The power (1-β error probability) was calculated by statistical test “Means: Wilcoxon–Mann–Whitney test (two groups)” at the significance level (α error probability, p = 0.05). Effect size *d* was determined from the mean and variance in each group. Correlation coefficients were determined for identification of related metabolic networks among compounds. Principal component analysis (PCA) was conducted using the SIMCA-P + software (Umetrics Inc., Umea, Sweden). Pearson’s correlation coefficients among metabolites were calculated using Microsoft Excel. The minimal value considered significant in the present study (n = 18) was 0.5. Heatmaps present standardized abundance data for each metabolite. T scores were calculated from the following formula: T score = [(sample peak area − average of population peak area) × 10/SD of population peak area] + 50. Therefore, the mean and SD are 50 and 10, respectively.

## Supplementary Information

Supplementary Information.

## Data Availability

Raw LC–MS data in mzML format are available in the MetaboLights repository (http://www.ebi.ac.uk/metabolights). Data for the 18 volunteers are available under accession number MTBLS6879.
