# Supplementary material for: Markers for obese and non-obese Type 2 diabetes identified using whole blood metabolomics
Source: Sci Rep. 2023 Feb 11;13:2460. doi: 10.1038/s41598-023-29619-4 (PMC9922320; doi:10.1038/s41598-023-29619-4)
Supplement: Supplementary file 1 — Supplementary Information. [file 41598_2023_29619_MOESM1_ESM.pdf]

Supplementary Information

Markers for obese and non-obese Type 2 diabetes identified using whole blood metabolomics

Takayuki Teruya[1], Sumito Sunagawa[2], Ayaka Mori[1], Hiroaki Masuzaki[2] and Mitsuhiro Yanagida[1*]

[1] G0 Cell Unit, Okinawa Institute of Science and Technology Graduate University, Okinawa, Japan

[2] Division of Endocrinology, Diabetes and Metabolism, Hematology, Rheumatology (Second Department of Internal Medicine), Graduate School of Medicine, University of the Ryukyus, Okinawa, Japan

*Correspondence: Mitsuhiro Yanagida (myanagid@gmail.com)

This file includes **Supplementary Tables S1-S3** and **Figures S1-S3**.

Supplementary Table S1. Characteristics of subjects. Individual data from 5 non-obese, non-diabetic, 5 obese, non-diabetic, 4 non-obese Type 2 diabetic, and 4 obese Type 2 diabetic subjects are shown. BMI, body mass index; PG, fasting plasma glucose.

group	Subject No.	Age	Gender	BMI (kg/m^2)	HbA1c (%)	PG (mg/dL)
non-Ob ND	1	78	F	21.1	5.5	82
	2	63	F	20.9	5.8	93
	3	63	M	20.7	5.3	88
	4	42	F	20.6	5.2	87
	5	44	M	20.6	5.2	81
Ob ND	6	69	M	29.6	5.4	102
	7	42	F	31.9	5.5	102
	8	41	M	29.1	5.1	93
	9	79	F	34.2	5.2	88
	10	69	F	31.4	5.8	101
non-Ob T2D	11	59	F	20.8	8.7	153
	12	68	M	23.1	9.5	108
	13	54	F	19.3	12.6	264
	14	52	M	20.2	9.3	223
Ob T2D	15	51	M	28.4	9.8	151
	16	39	F	35.8	7.2	115
	17	72	F	34.1	8.8	146
	18	56	M	31.0	6.9	149

Supplementary Table S2. Chromatographic data and mass spectra of 125 compounds. Compounds were identified using either commercially available standards (STD) or by analysis of MS/MS spectra (MS/MS), if no standard was available.

Compounds	Status	Formula	Ionization	Theoretical m/z	Detected m/z	m/z error (ppm)	RT (min)
1,5-Anhydroglucitol	STD	C6H12O5	[M-H]-	163.0612	163.0617	3.0	9.9
2-Hydroxybutyrate	STD	C4H8O3	[M-H]-	103.0401	103.0409	8.1	4.8
2-Oxoglutarate	STD	C5H6O5	[M-H]-	145.0142	145.0146	2.5	13.9
3-Hydroxybutyrate	STD	C4H8O3	[M-H]-	103.0401	103.0410	9.2	6.0
4-Aminobenzoate	STD	C7H7NO2	[M+H]+	138.0555	138.0545	-7.0	7.6
4-Guanidinobutanoate	STD	C5H11N3O2	[M+H]+	146.0930	146.0922	-5.1	14.7
6-Phosphogluconate	STD	C6H13O10P	[M-H]-	275.0174	275.0171	-0.8	17.3
Acetyl-carnitine	STD	C9H17NO4	[M+H]+	204.1236	204.1227	-4.6	9.1
Acetyl-carnosine	STD	C11H16N4O4	[M+H]+	269.1250	269.1238	-4.3	6.7
Adenine	STD	C5H5N5	[M+H]+	136.0623	136.0614	-6.7	7.0
Adenosine	STD	C10H13N5O4	[M+H]+	268.1046	268.1029	-6.2	6.7
ADP	STD	C10H15N5O10P2	[M+H]+	428.0372	428.0359	-3.2	14.8
Aminobutyrate	STD	C4H9NO2	[M-H]-	102.0561	102.0568	7.2	12.8
AMP	STD	C10H14N5O7P	[M+H]+	348.0709	348.0699	-2.9	12.8
Arginine	STD	C6H14N4O2	[M+H]+	175.1195	175.1185	-5.6	26.2
Asparagine	STD	C4H8N2O3	[M+H]+	133.0613	133.0601	-8.9	14.3
Aspartate	STD	C4H7NO4	[M+H]+	134.0453	134.0443	-7.9	13.9
ATP	STD	C10H16N5O13P3	[M+H]+	508.0036	508.0024	-2.3	16.1
Betaine	STD	C5H11NO2	[M+H]+	118.0868	118.0857	-9.5	9.3
Butyro-betaine	STD	C7H15NO2	[M+H]+	146.1181	146.1172	-5.9	12.5
(iso)Butyryl-carnitine	STD	C11H21NO4	[M+H]+	232.1549	232.1541	-3.4	6.4
Caffeine	STD	C8H10N4O2	[M+H]+	195.0882	195.0874	-3.9	4.1
Carnitine	STD	C7H15NO3	[M+H]+	162.1130	162.1119	-6.7	12.4
Carnosine	STD	C9H14N4O3	[M+H]+	227.1144	227.1136	-3.4	15.4
CDP-choline	STD	C14H26N4O11P2	[M+H]+	489.1152	489.1132	-3.9	15.1
CDP-ethanolamine	STD	C11H20N4O11P2	[M-H]-	445.0531	445.0536	1.0	16.1
Chenodeoxycholate	STD	C24H40O4	[M-H]-	391.2854	391.2850	-1.0	3.7
Citrate	STD	C6H8O7	[M-H]-	191.0197	191.0200	1.5	17.5
Citrulline	STD	C6H13N3O3	[M+H]+	176.1035	176.1026	-4.9	15.5
Creatine	STD	C4H9N3O2	[M-H]-	130.0622	130.0628	4.3	14.0
Creatinine	STD	C4H7N3O	[M-H]-	112.0516	112.0525	8.0	7.2
CTP	STD	C9H16N3O14P3	[M-H]-	481.9772	481.9778	1.2	17.7
Cytidine	STD	C9H13N3O5	[M+H]+	244.0933	244.0925	-3.5	10.1
Decanoyl-carnitine	STD	C17H33NO4	[M+H]+	316.2488	316.2478	-3.3	4.7
Dimethyl-arginine	STD	C8H18N4O2	[M+H]+	203.1508	203.1501	-3.4	22.2
Dimethyl-guanosine	STD	C12H17N5O5	[M+H]+	312.1308	312.1295	-4.1	6.3
Dimethyl-lysine	STD	C8H18N2O2	[M+H]+	175.1447	175.1438	-4.6	21.7
Dimethyl-proline	STD	C7H13NO2	[M+H]+	144.1025	144.1014	-7.0	8.4
Dimethyl-xanthine	STD	C7H8N4O2	[M+H]+	181.0726	181.0718	-4.4	4.8
Diphosphoglycerate	STD	C3H8O10P2	[M-H]-	264.9520	264.9516	-1.7	18.0
Dodecanoyl-carnitine	STD	C19H37NO4	[M+H]+	344.2801	344.2790	-3.1	4.5
Ergothioneine	STD	C9H15N3O2S	[M+H]+	230.0963	230.0953	-4.3	13.9
Fructose-1,6-diphosphate	STD	C6H14O12P2	[M-H]-	338.9888	338.9886	-0.5	17.8
Fructose-6-phosphate	STD	C6H13O9P	[M-H]-	259.0224	259.0225	0.1	15.4
GDP	STD	C10H15N5O11P2	[M-H]-	442.0171	442.0170	-0.2	17.6
GDP-glucose	STD	C16H25N5O16P2	[M-H]-	604.0699	604.0715	2.7	17.7
Gluconate	STD	C6H12O7	[M-H]-	195.0510	195.0510	0.0	12.4
Glucose-6-phosphate	STD	C6H13O9P	[M-H]-	259.0224	259.0226	0.7	16.5

Supplementary Table S2. (continued)

Compounds	Status	Formula	Ionization	Theoretical m/z	Detected m/z	m/z error (ppm)	RT (min)
Glutamate	STD	C5H9NO4	[M+H]+	148.0610	148.0600	-6.6	13.5
Glutamine	STD	C5H10N2O3	[M-H]-	145.0619	145.0622	2.3	14.3
Glutathione disulfide	STD	C20H32N6O12S2	[M+H]+	613.1598	613.1587	-1.7	17.3
Glyceraldehyde-3-phosphate	STD	C3H7O6P	[M-H]-	168.9907	168.9908	0.3	14.4
Glycerol-phosphate	STD	C3H9O6P	[M-H]-	171.0064	171.0068	2.5	14.1
Glycerophosphocholine	STD	C8H20NO6P	[M+H]+	258.1106	258.1095	-4.6	14.3
Glycerophosphoethanolamine	MS/MS	C5H14NO6P	[M-H]-	214.0486	214.0489	1.6	15.3
Glycochenodeoxycholate	STD	C26H43NO5	[M-H]-	448.3068	448.3066	-0.5	3.6
GTP	STD	C10H16N5O14P3	[M-H]-	521.9834	521.9839	1.0	18.6
Hexanoyl-carnitine	STD	C13H25NO4	[M+H]+	260.1862	260.1852	-3.6	5.2
Hippurate	STD	C9H9NO3	[M+H]+	180.0661	180.0653	-4.2	4.4
Histidine	STD	C6H9N3O2	[M+H]+	156.0773	156.0764	-5.9	13.7
Hypoxanthine	STD	C5H4N4O	[M+H]+	137.0463	137.0455	-5.9	7.6
IMP	STD	C10H13N4O8P	[M-H]-	347.0398	347.0404	1.6	15.1
Indoxyl-sulfate	STD	C8H7NO4S	[M-H]-	212.0023	212.0022	-0.6	4.6
Isoleucine	STD	C6H13NO2	[M-H]-	130.0874	130.0881	6.1	9.4
Keto(iso)leucine	STD	C6H10O3	[M-H]-	129.0557	129.0564	4.9	3.6
Ketovaline	STD	C5H8O3	[M-H]-	115.0401	115.0408	6.7	3.9
Kynurenine	STD	C10H12N2O3	[M+H]+	209.0926	209.0918	-4.0	8.8
Leucine	STD	C6H13NO2	[M-H]-	130.0874	130.0881	6.1	8.7
Lysine	STD	C6H14N2O2	[M-H]-	145.0983	145.0989	4.6	25.3
Malate	STD	C4H6O5	[M-H]-	133.0142	133.0149	5.0	14.7
Methionine	STD	C5H11NO2S	[M+H]+	150.0589	150.0580	-5.7	9.6
myo-Inositol	STD	C6H12O6	[M-H]-	179.0561	179.0562	0.5	16.5
N-Acetyl-aspartate	STD	C6H9NO5	[M-H]-	174.0408	174.0412	2.3	13.1
N-Acetyl-glucosamine	STD	C8H15NO6	[M+H]+	222.0978	222.0970	-3.5	10.2
N-Methyl-guanosine	STD	C11H15N5O5	[M+H]+	298.1151	298.1141	-3.5	8.6
N-Methyl-adenosine	STD	C11H15N5O4	[M+H]+	282.1202	282.1181	-7.6	12.4
N1-Methyl-histidine	STD	C7H11N3O2	[M+H]+	170.0930	170.0921	-5.2	11.3
N2-Acetyl-arginine	STD	C8H16O3N4	[M+H]+	217.1301	217.1291	-4.6	14.5
N2-Acetyl-lysine	STD	C8H16O3N2	[M+H]+	189.1239	189.1230	-4.9	14.8
N3-Methyl-histidine	STD	C7H11N3O2	[M+H]+	170.0930	170.0921	-5.1	12.0
N6-Acetyl-lysine	STD	C8H16O3N2	[M+H]+	189.1239	189.1230	-4.6	12.2
N6-Methyl-lysine	STD	C7H16N2O2	[M+H]+	161.1290	161.1281	-5.6	24.2
NAD+	STD	C21H27N7O14P2	[M+H]+	664.1169	664.1149	-3.1	13.8
NADP+	STD	C21H28N7O17P3	[M+H]+	744.0833	744.0817	-2.1	16.7
Nicotinamide	STD	C6H6N2O	[M+H]+	123.0558	123.0548	-8.6	5.1
O-Methyl-guanosine	STD	C11H15N5O5	[M+H]+	298.1151	298.1142	-3.1	7.6
Octanoyl-carnitine	STD	C15H29NO4	[M+H]+	288.2175	288.2166	-3.0	4.7
Ophthalmic acid	STD	C11H19N3O6	[M+H]+	290.1352	290.1342	-3.5	12.4
Ornithine	STD	C5H12N2O2	[M-H]-	131.0826	131.0835	6.7	23.2
Pantothenate	STD	C9H17NO5	[M+H]+	220.1185	220.1176	-3.9	5.2
Pentose-phosphate	STD	C5H11O8P	[M-H]-	229.0119	229.0120	0.6	15.0
Phenylalanine	STD	C9H11NO2	[M+H]+	166.0868	166.0859	-5.6	7.6
Phosphocholine	STD	C5H14NO4P	[M+H]+	184.0739	184.0731	-4.1	14.7
Phosphocreatine	STD	C4H10N3O5P	[M-H]-	210.0285	210.0289	1.5	14.6
Phosphoenolpyruvate	STD	C3H5O6P	[M-H]-	166.9751	166.9753	1.3	17.1
Phosphoethanolamine	STD	C2H8NO4P	[M-H]-	140.0118	140.0125	4.6	15.6
Phosphoglycerate	STD	C3H7O7P	[M-H]-	184.9857	184.9861	2.4	16.5
Proline	STD	C5H9NO2	[M+H]+	116.0712	116.0700	-10.0	11.4
Propionyl-carnitine	STD	C10H19NO4	[M+H]+	218.1392	218.1384	-4.0	7.5
Pseudouridine	STD	C9H12N2O6	[M-H]-	243.0623	243.0625	1.1	10.1

Supplementary Table S2. (continued)

Compounds	Status	Formula	Ionixation	Theoretical m/z	Detected m/z	m/z error (ppm)	RT (min)
S-Adenosyl-homocysteine	STD	C14H20N6O5S	[M+H]+	385.1294	385.1282	-3.1	13.1
S-Adenosyl-methionine	STD	C15H22N6O5S	[M+H]+	399.1451	399.1442	-2.1	17.1
S-Methyl-ergothioneine	STD	C10H17N3O2S	[M+H]+	244.1120	244.1109	-4.5	8.6
Sedoheptulose-7-phosphate	STD	C7H15O10P	[M-H]-	289.0330	289.0334	1.2	15.8
Serine	STD	C3H7NO3	[M+H]+	106.0504	106.0494	-10.0	14.9
Succinate	STD	C4H6O4	[M-H]-	117.0193	117.0199	4.7	13.8
Taurine	STD	C2H7NO3S	[M-H]-	124.0074	124.0078	3.4	13.1
Tetradecanoyl-carnitine	STD	C21H41NO4	[M+H]+	372.3114	372.3110	-1.1	4.1
Threonine	STD	C4H9NO3	[M+H]+	120.0661	120.0650	-8.6	13.3
Trimethyl-histidine	STD	C9H15N3O2	[M+H]+	198.1243	198.1234	-4.5	11.1
Trimethyl-lysine	STD	C9H20N2O2	[M+H]+	189.1603	189.1595	-4.4	22.9
Trimethyl-tryptophan	STD	C14H18N2O2	[M+H]+	247.1447	247.1437	-3.9	6.3
Trimethyl-tyrosine	MS/MS	C12H17NO3	[M+H]+	224.1287	224.1276	-5.0	8.1
Tryptophan	STD	C11H12N2O2	[M+H]+	205.0977	205.0969	-3.8	9.8
Tyrosine	STD	C9H11NO3	[M+H]+	182.0817	182.0809	-4.3	11.9
UDP-glucose	STD	C15H24N2O17P2	[M-H]-	565.0477	565.0487	1.7	15.7
UDP-glucuronate	STD	C15H22N2O18P2	[M-H]-	579.0270	579.0279	1.6	18.3
UDP-N-acetylglucosamine	STD	C17H27N3O17P2	[M-H]-	606.0743	606.0756	2.1	14.4
UMP	STD	C9H13N2O9P	[M-H]-	323.0286	323.0289	0.8	14.3
Urate	STD	C5H4N4O3	[M-H]-	167.0211	167.0210	-0.3	12.2
Uridine	STD	C9H12N2O6	[M-H]-	243.0623	243.0626	1.2	7.3
UTP	STD	C9H15N2O15P3	[M-H]-	482.9613	482.9621	1.8	17.2
(iso)Valeryl-carnitine	STD	C12H23NO4	[M+H]+	246.1705	246.1697	-3.3	5.7
Valine	STD	C5H11NO2	[M-H]-	116.0717	116.0726	8.0	11.0
Xanthine	STD	C5H4N4O2	[M-H]-	151.0261	151.0268	4.4	8.4

Supplementary Table S3. A list of 125 blood metabolites in 14 categories. Abundances of compounds are semi-quantitatively separated into 3 groups, H (high), M (medium), and L (low), by their peak areas. P-values were calculated using the Mann–Whitney U-test. Peak ratios were calculated using the median of peak abundance in non-diabetic and Type 2 diabetic or non-obese and obese.

Compounds	RBC-enriched	Peak abundance	T2D/non-T2D		Ob/non-Ob	
			p-value	Peak ratio	p-value	Peak ratio
Nucleotides (9)						
ATP	●	H	0.41		0.063	
ADP	●	H-M	0.41		0.063	
AMP	●	M	0.46		0.19	
GTP	●	M	0.90		0.44	
UMP	●	M-L	0.00055	3.32	0.93	
CTP		L	0.32		0.86	
GDP	●	L	0.76		0.86	
IMP	●	L	0.15		0.39	
UTP		L	0.083		0.67	
Nucleosides, nucleobases, and derivatives (14)						
Caffeine		H-L	0.0085	0.14	0.30	
Dimethyl-xanthine		H-L	0.0044	0.16	0.67	
Urate	●	M	0.51		0.0040	1.26
N-Methyl-adenosine		M-L	0.0014	2.70	0.39	
Cytidine		M-L	0.000046	0.60	0.44	
Pseudouridine		M-L	0.12		0.014	1.11
Uridine		L	0.15		1.00	
Xanthine		L	0.36		0.040	1.42
Adenine	●	L	0.034	1.79	0.19	
Adenosine		L	0.24		0.93	
Dimethyl-guanosine		L	0.083		0.11	
N-Methyl-guanosine		L	0.016	0.55	0.30	
O-Methyl-guanosine		L	0.12		0.39	
Hypoxanthine		L	0.27		0.019	1.31
Nucleotide-sugar derivatives (4)						
UDP-glucose	●	M-L	0.0031	0.53	0.49	
UDP-N-acetylglucosamine	●	M-L	0.41		0.93	
UDP-glucuronate	●	L	0.000046	0.45	0.60	
GDP-glucose	●	L	0.20		0.22	
Sugar phosphates (11)						
Diphosphoglycerate	●	H-M	0.76		1.00	
Fructose-1,6-diphosphate	●	M	0.32		1.00	
Fructose-6-phosphate	●	L	0.00055	1.39	1.00	
6-Phosphogluconate	●	L	0.00055	2.10	0.86	
Glucose-6-phosphate	●	L	0.12		0.60	
Glyceraldehyde-3-phosphate	●	L	0.0014	0.14	0.93	
Glycerol-phosphate	●	L	0.27		1.00	
Pentose-phosphate	●	L	0.00018	0.33	0.44	
Phosphoenolpyruvate	●	L	0.012	1.57	0.19	
Phosphoglycerate	●	L	0.034	1.41	0.49	
Sedoheptulose-7-phosphate	●	L	0.0044	1.50	0.86	

Supplementary Table S3. (continued)

Compounds	RBC-enriched	Peak abundance	T2D/non-T2D		Ob/non-Ob	
			p-value	Peak ratio	p-value	Peak ratio
Sugar derivatives (4)						
N-Acetyl-glucosamine	●	M	0.0044	1.32	0.16	
1,5-Anhydroglucitol		M-L	0.0031	0.27	0.44	
Gluconate	●	M-L	0.76		0.80	
myo-Inositol		L	0.27		0.22	
Vitamins and coenzymes (5)						
NAD+	●	H-M	0.17		0.44	
Nicotinamide	●	H-M	0.12		0.80	
4-Aminobenzoate		M-L	0.69		0.42	
Pantothenate	●	M-L	0.76		0.077	
$NADP^+$	●	L	0.043	1.39	0.040	1.40
Organic acids (9)						
Glycochenodeoxycholate		H-L	0.17		0.19	
Chenodeoxycholate		M-L	0.15		0.39	
Aminobutyrate	●	L	0.10		0.040	1.30
2-Hydroxybutyrate		L	0.0085	2.01	0.30	
2-Oxoglutarate		L	0.27		0.031	1.81
3-Hydroxybutyrate		L	0.10		0.014	1.58
Citrate		L	0.32		0.80	
Malate	●	L	0.32		0.011	1.22
Succinate	●	L	0.016	1.48	0.49	
Antioxidants (2)						
Glutathione disulfide	●	H	0.068		0.050	
Ergothioneine	●	H	0.76		0.39	
Standard amino acids (17)						
Arginine		H	0.068		0.063	
Phenylalanine		H	0.055		0.014	1.33
Proline		H	0.76		0.0078	1.27
Glutamate		H	0.32		0.14	
Histidine		H	0.41		0.040	1.25
Tryptophan		H	0.10		0.0056	1.25
Threonine		H-M	0.0085	0.67	0.050	
Tyrosine		H-M	0.46		0.0056	1.37
Methionine		H-M	0.016	0.75	0.094	
Glutamine		M	0.24		0.73	
Leucine		M	0.12		0.11	
Serine		M	0.0014	0.70	0.050	
Lysine		M-L	1.00		0.86	
Asparagine		M-L	0.083		0.040	1.30
Aspartate		M-L	0.57		0.30	
Isoleucine		M-L	0.15		0.11	
Valine		L	0.32		0.11	
Acetylated amino acids (4)						
N-Acetyl-aspartate		L	0.027	0.71	0.73	
N2-Acetyl-arginine		L	0.32		0.019	1.76
N2-Acetyl-lysine		L	0.46		0.0019	1.61
N6-Acetyl-lysine		L	0.83		0.73	

Supplementary Table S3. (continued)

Compounds	RBC-enriched	Peak abundance	T2D/non-T2D		Ob/non-Ob	
			p-value	Peak ratio	p-value	Peak ratio
Methylated amino acids (13)						
Betaine		H	0.90		0.019	1.05
Butyro-betaine	●	H-M	0.24		0.077	
Dimethyl-proline	●	H-M	0.043	2.42	0.86	
Trimethyl-lysine	●	H-M	0.46		0.011	1.80
Trimethyl-tryptophan	●	H-M	0.15		0.80	
Dimethyl-arginine		M	0.20		0.0019	1.48
N6-Methyl-lysine		M	0.32		0.67	
S-Methyl-ergothioneine	●	M	0.90		0.39	
Dimethyl-lysine		M-L	0.055		0.16	
N1-Methyl-histidine		M-L	0.27		0.040	2.08
N3-Methyl-histidine		M-L	0.055		0.86	
Trimethyl-tyrosine	●	M-L	0.32		0.60	
Trimethyl-histidine	●	L	0.97		0.094	
Other amino acids (17)						
Citrulline		H	0.016	0.75	0.040	1.20
Hippurate		H-L	0.17		0.024	2.41
Taurine		M	0.0044	0.79	0.80	
Acetyl-carnosine		M-L	0.083		0.11	
Creatine	●	M-L	0.63		0.49	
Creatinine		M-L	0.27		0.16	
Indoxyl-sulfate		M-L	0.055		0.80	
Keto(iso)leucine		M-L	0.083		0.077	
Kynurenine		M-L	0.0014	0.50	0.22	
Ophthalmic acid		M-L	0.41		0.60	
4-Guanidinobutanoate		L	0.51		0.14	
Carnosine	●	L	0.36		0.063	
Ketovaline		L	0.97		0.019	1.57
Ornithine		L	0.12		0.93	
Phosphocreatine	●	L	0.46		0.44	
S-Adenosyl-homocysteine	●	L	0.46		0.11	
S-Adenosyl-methionine	●	L	0.016	0.52	0.11	
Lipid metabolites (6)						
Glycerophosphocholine		H-M	0.46		0.86	
Phosphocholine		H-M	0.90		0.031	1.49
CDP-choline		M-L	0.46		0.024	1.66
Glycerophosphoethanolamine		L	0.012	0.82	0.44	
Phosphoethanolamine		L	0.57		0.49	
CDP-ethanolamine		L	0.17		0.094	
Carnitines (10)						
Carnitine		H	0.90		0.0056	1.24
Acetyl-carnitine	●	H	1.00		0.0012	1.29
Propionyl-carnitine	●	H-M	0.70		0.00049	2.64
(iso)Butyryl-carnitine		M	0.70		0.00078	2.04
Decanoyl-carnitine		M	0.083		0.30	
(iso)Valeryl-carnitine		M-L	0.90		0.0019	1.76
Hexanoyl-carnitine		M-L	0.63		0.11	
Octanoyl-carnitine		M-L	0.36		0.44	
Dodecanoyl-carnitine		M-L	0.043	0.58	0.26	
Tetradecanoyl-carnitine	●	L	0.27		0.19	

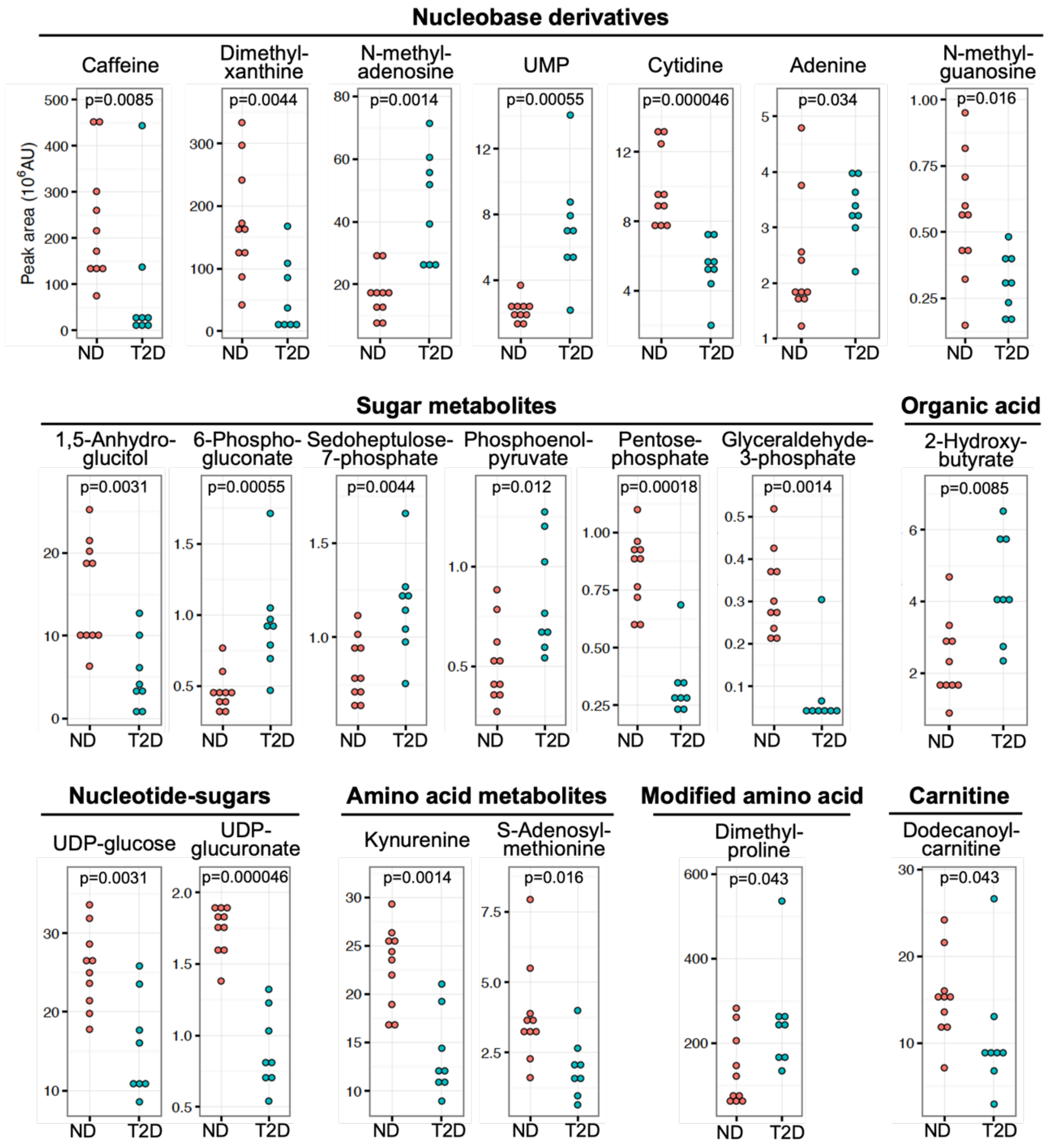

Supplementary Figure S1. Dot plot profiling of Type 2 diabetes marker abundances.

Peak areas of the non-diabetic and Type 2 diabetic groups are plotted. Eight of 20 compounds increased significantly and 12 compounds decreased. Distributions of cytidine and UDP-glucuronate were completely segregated between non-diabetic and Type 2 diabetic.

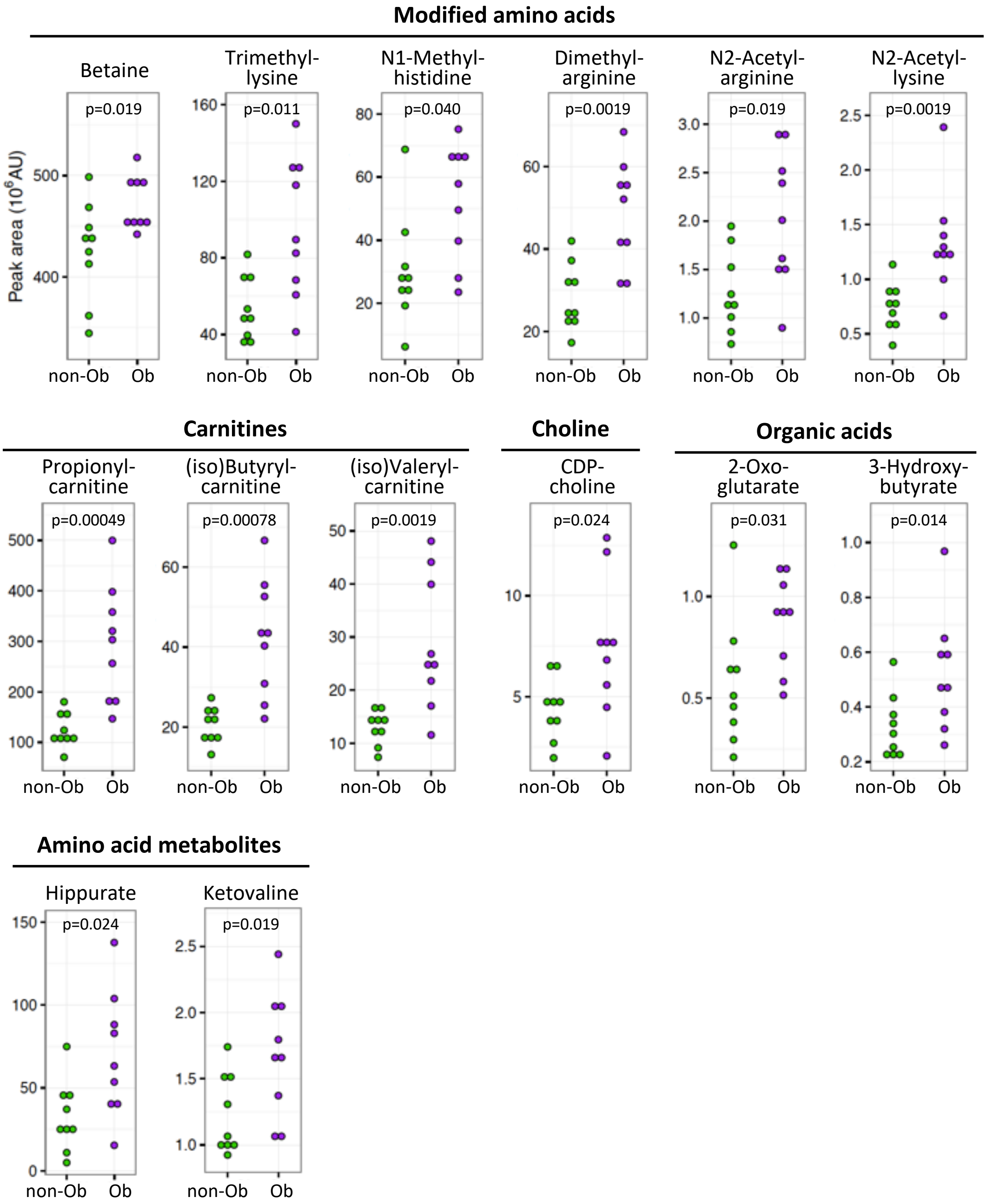

Supplementary Figure S2. Dot plot profiling of 13 obesity marker abundances. Peak areas of the non-obese group and obese group are plotted. All 13 compounds increased significantly in obesity. Of these, three carnitines showed the most significant differences.

Group	Correlation coefficient *r* (n=18)	HbA1c	Plasma glucose	BMI	Dimethyl-proline	Phosphoenolpyruvate	Adenine	Sedoheptulose-7-phosphate	2-Hydroxybutyrate	UMP	6-Phosphogluconate	N-Methyl-adenosine	UDP-glucuronate	Glyceraldehyde-3-phosphate	UDP-glucose	Pentose-phosphate	Kynurenine	Cytidine	Caffeine	Dimethyl-xanthine	1,5-Anhydroglucitol	N-Methyl-guanosine	S-Adenosyl-methionine	Dodecanoyl-carnitine	N2-Acetyl-lysine	Dimethyl-arginine	Trimethyl-lysine	N1-Methyl-histidine	CDP-choline	Propionyl-carnitine	(iso)Butyryl-carnitine	(iso)Valeryl-carnitine	N2-Acetyl-arginine	2-Oxoglutarate	Ketovaline	Hippurate
					T2D-related																			Obese-related												
	Plasma glucose ↑	-0.15																																		
	BMI ↑	-0.20	0.70																																	
T2D-related (20)	Dimethyl-proline ↑	0.49	-0.09	-0.01																																
	Phosphoenolpyruvate ↑	0.53	0.05	0.16	0.59																															
	Adenine ↑	0.28	-0.13	0.28	0.35	0.45																														
	Sedoheptulose-7-phosphate ↑	0.47	-0.05	0.05	0.25	0.33	0.54																													
	2-Hydroxybutyrate ↑	0.47	0.17	0.27	0.30	0.32	0.32	0.31																												
	UMP ↑	0.75	-0.01	-0.03	0.69	0.48	0.40	0.59	0.44																											
	6-Phosphogluconate ↑	0.74	-0.07	-0.22	0.29	0.47	0.41	0.62	0.35	0.75																										
	N-Methyl-adenosine ↑	0.79	-0.02	0.01	0.38	0.50	0.40	0.44	0.47	0.80	0.83																									
	UDP-glucuronate ↓	-0.75	-0.01	-0.14	-0.53	-0.52	-0.59	-0.67	-0.59	-0.89	-0.76	-0.77																								
	Glyceraldehyde-3-phosphate ↓	-0.77	0.07	0.00	-0.40	-0.45	-0.53	-0.61	-0.38	-0.71	-0.64	-0.67	0.82																							
	Pentose-phosphate ↓	-0.78	-0.02	-0.18	-0.47	-0.48	-0.63	-0.58	-0.48	-0.72	-0.64	-0.74	0.84	0.92																						
	UDP-glucose ↓	-0.65	0.22	0.02	-0.14	-0.26	-0.46	-0.29	-0.20	-0.34	-0.45	-0.56	0.55	0.77	0.80																					
	Kynurenine ↓	-0.83	0.26	0.31	-0.36	-0.40	-0.23	-0.47	-0.47	-0.74	-0.69	-0.77	0.72	0.80	0.72	0.54																				
	Cytidine ↓	-0.80	0.18	0.17	-0.25	-0.38	-0.17	-0.45	-0.42	-0.53	-0.57	-0.52	0.68	0.79	0.69	0.69	0.70																			
	Caffeine ↓	-0.56	0.09	0.13	-0.22	-0.07	-0.20	-0.38	-0.20	-0.51	-0.44	-0.39	0.55	0.82	0.70	0.57	0.65	0.75																		
	Dimethyl-xanthine ↓	-0.58	-0.10	-0.04	-0.13	-0.16	-0.04	-0.37	-0.35	-0.49	-0.37	-0.41	0.59	0.80	0.69	0.56	0.70	0.75	0.83																	
	1,5-Anhydroglucitol ↓	-0.75	0.25	0.15	-0.48	-0.33	-0.40	-0.38	-0.44	-0.56	-0.59	-0.65	0.54	0.50	0.70	0.57	0.56	0.51	0.39	0.24																
	N-Methyl-guanosine ↓	-0.48	0.51	0.32	-0.17	-0.25	-0.41	-0.62	-0.34	-0.40	-0.48	-0.43	0.47	0.54	0.49	0.38	0.70	0.42	0.35	0.30	0.52															
	S-Adenosyl-methionine ↓	-0.59	0.11	0.25	-0.11	0.12	0.26	-0.16	-0.36	-0.47	-0.32	-0.46	0.45	0.47	0.40	0.42	0.65	0.58	0.46	0.70	0.30	0.24														
	Dodecanoyl-carnitine ↓	-0.58	0.38	0.32	-0.22	0.10	-0.02	-0.26	-0.12	-0.51	-0.37	-0.48	0.44	0.49	0.36	0.38	0.60	0.47	0.51	0.39	0.30	0.34	0.68													
Obese-related (13)	N2-Acetyl-lysine ↑	-0.28	0.44	0.54	0.08	0.45	0.14	0.09	0.31	-0.13	-0.14	-0.10	0.08	0.27	0.13	0.40	0.26	0.31	0.47	0.27	0.13	0.02	0.52	0.73												
	Dimethyl-arginine ↑	-0.41	0.71	0.63	-0.04	0.28	0.02	-0.08	-0.01	-0.17	-0.23	-0.15	0.16	0.22	0.18	0.38	0.33	0.45	0.39	0.22	0.42	0.25	0.52	0.63	0.78											
	Trimethyl-lysine ↑	-0.17	0.37	0.43	0.09	0.44	0.17	0.21	0.16	0.13	0.00	0.08	-0.06	0.13	0.14	0.46	0.05	0.29	0.37	0.27	0.26	-0.10	0.43	0.31	0.74	0.76										
	N1-Methyl-histidine ↑	-0.38	0.33	0.39	0.01	0.29	0.19	0.20	-0.10	-0.08	-0.19	-0.15	0.15	0.13	0.19	0.40	0.24	0.36	0.28	0.27	0.38	0.01	0.57	0.43	0.66	0.76	0.84									
	CDP-choline ↑	-0.14	0.35	0.33	0.16	0.37	0.09	-0.09	0.10	0.14	-0.03	0.22	0.01	0.13	0.09	0.34	-0.06	0.44	0.39	0.23	0.21	0.00	0.29	0.27	0.59	0.73	0.79	0.61								
	Propionyl-carnitine ↑	0.00	0.31	0.51	0.36	0.59	0.56	0.28	0.30	0.33	0.16	0.28	-0.30	-0.09	-0.16	0.20	-0.02	0.21	0.23	0.25	0.00	-0.08	0.48	0.22	0.64	0.63	0.86	0.73	0.73							
	(iso)Butyryl-carnitine ↑	0.03	0.38	0.57	0.31	0.53	0.51	0.33	0.26	0.35	0.18	0.30	-0.32	-0.14	-0.23	0.10	0.00	0.08	0.10	0.16	-0.09	-0.08	0.47	0.30	0.65	0.64	0.81	0.71	0.64	0.92						
	(iso)Valeryl-carnitine ↑	-0.24	0.18	0.54	0.01	0.48	0.59	0.22	0.15	-0.15	-0.10	-0.09	-0.03	0.03	-0.05	0.08	0.25	0.20	0.30	0.31	0.11	-0.12	0.72	0.56	0.74	0.61	0.68	0.71	0.44	0.77	0.76					
	N2-Acetyl-arginine ↑	-0.23	0.33	0.44	0.21	0.33	0.16	0.06	0.09	0.01	-0.19	-0.02	0.09	0.14	0.06	0.25	0.25	0.20	0.22	0.28	0.03	0.10	0.51	0.49	0.69	0.64	0.67	0.79	0.51	0.67	0.80	0.65				
	2-Oxoglutarate ↑	-0.47	0.40	0.49	-0.10	-0.03	0.01	-0.05	0.11	-0.31	-0.25	-0.19	0.29	0.49	0.29	0.43	0.45	0.63	0.59	0.48	0.16	0.09	0.49	0.53	0.69	0.62	0.44	0.32	0.49	0.35	0.36	0.43	0.34			
	Ketovaline ↑	-0.15	0.37	0.49	-0.20	0.11	0.11	-0.15	0.44	-0.24	-0.15	0.08	0.10	0.17	0.03	0.09	0.05	0.34	0.39	0.12	0.09	-0.03	0.14	0.29	0.55	0.50	0.36	0.15	0.57	0.34	0.23	0.39	0.09	0.70		
	Hippurate ↑	-0.10	0.25	0.36	0.37	0.18	0.17	-0.11	-0.08	0.18	-0.11	0.11	0.05	0.21	0.12	0.32	0.25	0.39	0.29	0.50	0.02	0.30	0.40	-0.08	0.19	0.35	0.52	0.47	0.50	0.67	0.61	0.29	0.53	0.23	0.05	
	3-Hydroxybutyrate ↑	0.08	0.43	0.48	0.08	0.14	0.42	0.37	0.64	0.15	0.16	0.09	-0.32	-0.07	-0.15	0.09	0.09	-0.06	0.09	0.13	-0.12	-0.06	0.18	0.09	0.40	0.28	0.41	0.25	0.08	0.51	0.50	0.47	0.27	0.33	0.34	0.28

Supplementary Figure S3. Correlation analysis of 20 Type 2 diabetes and 13 obesity markers. Pearson's correlation analysis for 18 subjects. Positive (r > 0.5) and negative (r < -0.5) correlations are shown in orange and green cells, respectively (see text). Arrows indicate either an increase↑ or a decrease↓ of metabolite levels in comparison with each control.
